# Comparative Performance of ChatGPT 3.5 and GPT4 on Rhinology Standardized Board Examination Questions

**DOI:** 10.1002/oto2.164

**Published:** 2024-06-27

**Authors:** Evan A. Patel, Lindsay Fleischer, Peter Filip, Michael Eggerstedt, Michael Hutz, Elias Michaelides, Pete S. Batra, Bobby A. Tajudeen

**Affiliations:** ^1^ Department of Otorhinolaryngology–Head and Neck Surgery Rush University Medical Center Chicago Illinois USA

**Keywords:** artificial intelligence, machine learning, rhinology

## Abstract

**Objective:**

Advances in deep learning and artificial intelligence (AI) have led to the emergence of large language models (LLM) like ChatGPT from OpenAI. The study aimed to evaluate the performance of ChatGPT 3.5 and GPT4 on Otolaryngology (Rhinology) Standardized Board Examination questions in comparison to Otolaryngology residents.

**Methods:**

This study selected all 127 rhinology standardized questions from www.boardvitals.com, a commonly used study tool by otolaryngology residents preparing for board exams. Ninety‐three text‐based questions were administered to ChatGPT 3.5 and GPT4, and their answers were compared with the average results of the question bank (used primarily by otolaryngology residents). Thirty‐four image‐based questions were provided to GPT4 and underwent the same analysis. Based on the findings of an earlier study, a pass‐fail cutoff was set at the 10th percentile.

**Results:**

On text‐based questions, ChatGPT 3.5 answered correctly 45.2% of the time (8th percentile) (*P* = .0001), while GPT4 achieved 86.0% (66th percentile) (*P* = .001). GPT4 answered image‐based questions correctly 64.7% of the time. Projections suggest that ChatGPT 3.5 might not pass the American Board of Otolaryngology Written Question Exam (ABOto WQE), whereas GPT4 stands a strong chance of passing.

**Discussion:**

The older LLM, ChatGPT 3.5, is unlikely to pass the ABOto WQE. However, the advanced GPT4 model exhibits a much higher likelihood of success. This rapid progression in AI indicates its potential future role in otolaryngology education.

**Implications for Practice:**

As AI technology rapidly advances, it may be that AI‐assisted medical education, diagnosis, and treatment planning become commonplace in the medical and surgical landscape.

**Level of Evidence:**

Level 5.

Over the past decade, advances in machine learning, deep learning, and artificial intelligence (AI) have changed the way humans approach a wide variety of tasks and industries, ranging from manufacturing to consumer products. Deep learning from neural networks has implications for improving surgical and clinical precision and accuracy, patient education, data interpretation, information management, and many other potential applications within subspecialty care.^1^, ^2^, ^3^, ^4^, ^5^, ^6^ Although these recent developments have made substantial contributions, their use requires considerable time, effort, and data specific to that area of interest. These types of AI can generally considered to be domain specific. Because their training data are specific, their tasks and functions are also specific, meaning they cannot perform other functions outside of their expertise. Therefore, these types of AI cannot be considered generalizable or multifunctional.

AI has become an increasingly important tool for medical education as well as providing fast access to many years of data and includes computer‐based models, virtual reality simulations, and personalized learning platforms.^5^, ^7^, ^8^ As the capabilities of AI continue their rapid advance, it is becoming increasingly important to regularly evaluate the competency of AI‐powered tools. This evaluation is crucial to maintain high standards and prevent potential errors or biases, especially when addressing generative AI models that may demonstrate flawed reasoning or deliver misinformation that could harm patients or spread inaccurate information. Given the relatively limited understanding of these large language model's (LLM's) abilities in the domain of otolaryngology knowledge, it is especially important to assess the accuracy of AI‐powered tools in this field. By doing so, we can identify any shortcomings or areas for improvement and optimize the benefits of AI technology for health care providers and patients alike.

This study therefore sought to answer what percentage of standardized rhinology‐related board exam questions could a generative, pretrained transformer chatbot (ChatGPT 3.5 and GPT4) answer correctly. Given that a score lower than the 10th percentile corresponds with a high likelihood of failing the American Board of Otolaryngology Written Question Exam (ABOto WQE), we sought to compare performance against resident average scores and ultimately determine whether either of these LLM's was likely to pass the ABOto WQE.^9^ Finally, we aimed to explore whether increasing question difficulty impacted either of the LLM's abilities to select the correct answer choices.

## Methods

### Study Design and Setting

This was an experimental study using a commercially available LLM called ChatGPT 3.5 and a newer model hidden behind a paywall called ChatGPT4. These LLM's utilize self‐attention mechanisms and large amounts of training data to generate natural language responses to input text in conversational context. They are especially effective at handling long‐range dependencies and creating coherent and contextually appropriate responses. Self‐attention mechanisms are often used in natural language processing tasks such as language translation and text generation, where the model must understand the relationships between words in a sentence or a document. Long‐range dependencies refer to the relationship between distant parts of a sequence of input data or text, and combined with self‐attention allow accurate understanding and meaning of sentences that generate appropriate responses. However, one large improvement to ChatGPT4 over ChatGPT 3.5 is the ability to utilize open‐source plugins to accomplish tasks. For example, both LLM's are server contained, meaning that they cannot independently access data from the internet or perform search functions for new information. All responses are generated based on the abstract relationship between words in the neural network. However, with the introduction of plugins, users can augment ChatGPT4 to access the internet, read PDF's, and analyze images.

### Question Set and Testing

A total of 127 rhinology‐related standardized questions based on the ABOto WQE were initially selected for use in this study. Questions were generated from www.boardvitals.com, which is a “medical specialty board certification preparation firm which was founded in 2012, offering study material and question banks for physicians, medical students, and others in the health‐care industry.” Because the LLM chatbot ChatGPT 3.5 is purely a text‐based input program, questions that contained nontext‐based data could not be entered into the program, and the chatbot was unable to analyze or interpret imaging, figure, or picture‐based input data. A total of 26.8% (34 of 127) of the questions were excluded because they contained images, figures, tables, or charts, leaving 93 questions to be administered to the LLM's. The 34 image‐based questions were provided to GPT 4 for comparison against the resident average. All question stems and answer choices were entered verbatim into ChatGPT 3.5 text box, ensuring no duplicate questions were used, and the LLM was prompted to select the best answer. To reduce any memory retention bias, a new chat session was administered for each question. Memory retention via recurrent neural networks can occur when the LLM learns new information and subsequently applies the data to future data inputs and outputs. This same methodology was applied to ChatGPT4 and responses from both LLM's were recorded and compared to the correct answer.

### Primary and Secondary Study Outcomes

The primary study outcome was to ascertain the percentage of questions the LLM would answer correctly and to determine whether there would be a difference in performance between ChatGPT 3.5 and the newer, more advanced model, ChatGPT4.

The secondary study outcome was to compare its performance to that of otolaryngology residents in order to ascertain whether either of the LLM's would perform well enough to pass the ABOto WQE, and to determine whether the LLM's performance would decrease as question difficulty increased. To estimate whether it is likely that the LLM could pass the ABOto WQE written examination, this study utilized previous study data suggesting the bottom 10th percentile of exam‐takers failed the WQE.^9^ Finally, to answer the question about the LLM's performance against increasingly challenging questions, this study examined the individual scoring of each LLM on questions rated “easy,” “medium,” or “hard” on www.boardvitals.com. Of the 93 questions, 41.9% (39 of 93) were ranked as “easy,” 33.3% (31 of 93) were considered “moderate,” and 24.7% (23 of 93) were ranked as “hard.” The performance of each LLM was evaluated as a percentage of correct answers at each of those ranked levels. Institutional Review Board approval was not required as no human subject or patient data was utilized.

### Statistical Analysis

A *χ* ^2^ test was used to ascertain whether the LLM's percentage of correct answers was different according to question difficulty, and a *P* value of <.05 was considered significant. The standard deviation for the data set was calculated and a standardized bell curve was generated. *Z* scores were calculated to identify the percentile that each LLM's score would place it into. Independent two‐sample *t* tests were used to identify statistical significance between each LLM and the average resident score; a *P* value of <.05 was considered significant.

## Results

Both ChatGPT 3.5 and ChatGPT4 were able to successfully interpret the question stem and answer choices, and provide a response. There were no glitches or issues encountered during the data collection process.

### Percentage of Rhinology‐Related Standardized Board Questions Answered Correctly

**Figure 1:**
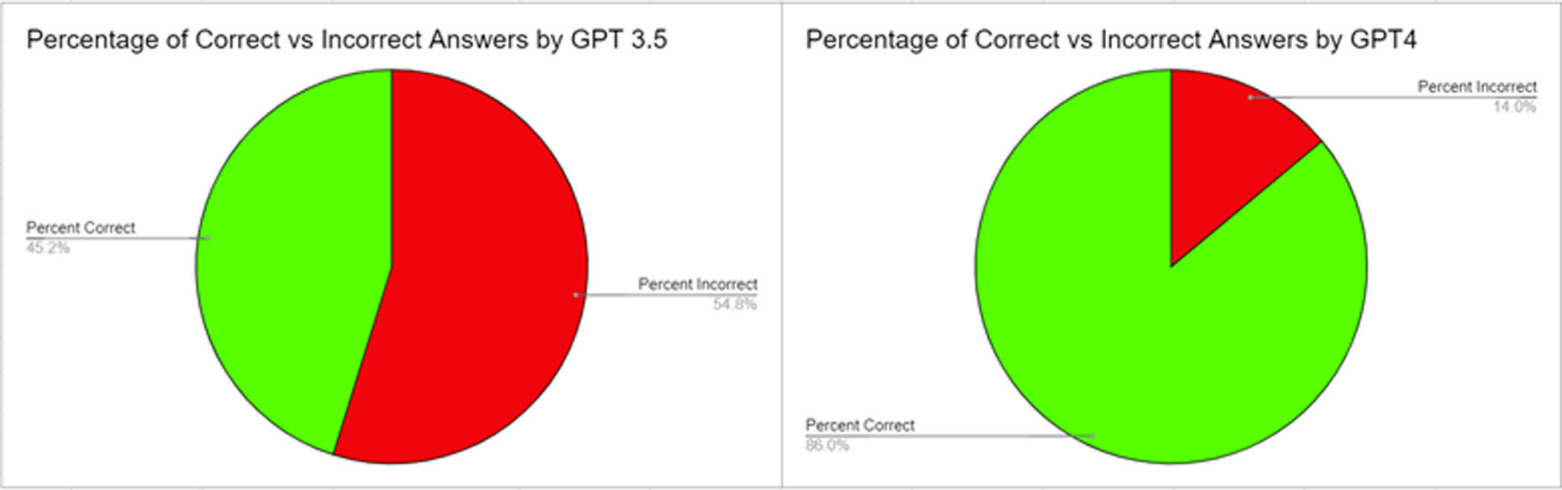
The chart on the left demonstrates the percentage of questions answered correctly (green) versus the percent incorrect (red) by OpenAI's ChatGPT 3.5. The chart on the right demonstrates the percentage of questions answered correctly (green) versus the percent incorrect (red) by OpenAI's ChatGPT4.

ChatGPT 3.5 selected the correct answer on text‐based questions 45.2% (42 of 93) of the time and 54.8% (51 of 93) of the time it answered incorrectly (Figure 1). ChatGPT4 selected the correct answer for text‐based questions 86.0% (80 of 93) of the time and 14.0% (13 of 93) of the time it answered incorrectly (Figure 1). For image‐based questions, GPT4 selected the correct answer 64.7% (22 of 34) of the time. Using the entire 127‐question set of text‐based and image‐based questions, GPT4 selected the correct answer 80.3% (102 of 127) of the time.

### Performance Comparison With Otolaryngology Residents

The average percentage correct on the 93‐question set by all Board Vitals users (predominantly residents) was 76.34% with a standard deviation of 22.63. This does not account for users retaking questions or utilizing other resources to aid in their question answering, so in reality, the average percentage correct by residents on this question set may be lower. Based on the predefined threshold of the 10th percentile of scores as a passing grade, ChatGPT 3.5 would likely not have passed the ABOto WQE.

GPT4 outperformed the average percent correct on the 93‐question data set. Based on the predefined threshold of the 10th percentile of scores as a passing grade, GPT4 would be very likely to pass the ABOto WQE.

In examining the performance outcomes of text‐based questions, statistical analyses revealed significant differences between the test scores of the LLM's and the resident average score. The comparison between ChatGPT 3.5's score (45.16%) and the average resident score (76.34%) yielded a highly significant difference, with *P *< .0001. The test scores of GPT4 (86.02%) and the resident average (76.34%) also differed significantly, although to a lesser extent (*P* = .010). These results suggest that the performance between the groups is not equivalent, with GPT4 demonstrating a statistically higher score compared to both ChatGPT 3.5 and the average resident score.

On image‐based questions, GPT4 scored a 64.7%, while the resident average on the 34 image‐based questions was a 76.5%. When looking at the combined performance on the combined 127 image and text‐based questions, the resident average was 76.38%, while GPT4 scored 80.3% (*P* = .431).

### Performance in Relation to Increasingly Difficult Question Levels

ChatGPT 3.5's performance decreased as text‐based question difficulty level increased with the LLM answering 58.97% (23 of 39) of “easy” questions, 45.16% (14 of 31) “moderate” questions, and 21.74% (5 of 23) “hard” questions correctly; (*P* = .109) (Table 1). This data analysis demonstrates a trend but did not reach statistical significance.

**Table 1:** This Table Demonstrates the Number of Correct Answers Selected by OpenAI's ChatGPT 3.5 and ChatGPT4 Based on Question Difficulty (Ranked by Board Vitals as “easy,” “moderate,” or “hard”)

Difficulty level	Correct answers	Total questions	Percentage, %
ChatGPT 3.5			
Easy	23	39	58.97
Moderate	14	31	45.16
Hard	5	23	21.74
ChatGPT4			
Easy	38	39	97.44
Moderate	28	31	90.32
Hard	11	23	47.83

ChatGPT4's performance also decreased as the question difficulty level increased with the updated version answering 97.44% (38 of 39) of “easy” questions, 90.32% (28 of 31) of “moderate” questions, and 47.83% (11 of 23) of “hard” questions correctly; (*P* = .099) (Table 1). This data analysis also demonstrates a trend but did not reach statistical significance.

## Discussion

LLMs, a type of machine learning, use vast amounts of text to analyze and synthesize its responses more naturally. It is also considered to be a nondomain or few‐shot scenario, meaning a small amount of training data is utilized to execute that specific function, but the LLM can understand the request and process, analyze, and possibly use reasoning and chain of thought abilities to answer a broad range of questions.

Steps that occur when an LLM receives input data or a query are the following: (1) *Input embedding*: The relationship between the words is analyzed through a dense vector representation. (2) *Multiheaded self‐attention*: The transformer block uses multiheaded self‐attention to focus on varying parts of the inputs and understand their relationships. (3) *Feed‐forward network*: Output from self‐attention goes through a feed‐forward neural network to create a new abstract understanding by using complex mathematical functions to capture intricate patterns and relationships. (4) *Normalization and residual connections*: A deep neural network is created by repeating the normalization and residual connection components to process long text sequences and generate high‐quality outputs for language tasks such as text generation, question answering, and translation.^4^

A new AI model using LLMs and nonspecific domain areas, called ChatGPT (OpenAI), has gained recent attention with its novel way of processing information. OpenAI has been rapidly improving its LLM offerings, with the first iteration of this product, ChatGPT 1, created in June 2018. ChatGPT was trained on a data set referred to as “Common Crawl,” a publicly available set of billions of web pages. This is one of the largest text data sets in existence. By February 2019, ChatGPT 2 was introduced, with ChatGPT 3 following shortly afterward in June 2020. This was the first model made publicly available in March 2022, with ChatGPT 3.5 replacing ChatGPT 3 as a free public offering. One year later, in March 2023, OpenAI released their most powerful LLM yet, ChatGPT4, available for purchase. Within a relatively short technological timespan (5 years), the processing power and training behind these LLM's has dramatically improved, with ChatGPT4 demonstrating 10 times the processing power of ChatGPT 3.5 with only 1 year of added development. This rapid advancement has exciting implications for the future of our technological landscape. As newer AI tools continue to be rapidly developed, the competency of these tools must be regularly checked, evaluated, and updated.

ChatGPT 3.5, an LLM chatbot released less than two years ago, answered nearly half of the rhinology WQE‐based questions correctly. This LLM scored approximately in the eighth percentile (1.38 standard deviations below the average user of the data set), meaning that it seems unlikely that this LLM would be able to pass the written board certification examination. The reason for this poorer performance relative to mid‐level and upper‐level residents was likely due to the LLM performing more poorly as the complexity of the test questions increased. This suggests the model may have limitations in terms of its ability to integrate, synthesize, generalize, and apply factual knowledge in more‐nuanced ways.

ChatGPT4, an LLM released only 1 year after ChatGPT 3.5, was able to answer 86% of questions correctly, demonstrating the rapid technological development in the field of AI. A score this high places it in the 66th percentile of Board Vitals users (assuming a standardized curve), indicating that ChatGPT4 could potentially pass the written board certification examination. This also demonstrates the rapid improvement of newer iterations of AI models. Within 1 year of development, AI has made the leap from performing worse than resident physicians to outperforming them on this set of standardized questions. This highlights the accelerating pace of AI's potential capability to outperform highly trained humans in specific tasks such as standardized testing.

There are likely to be practical advantages to and applications of AI within this context. One benefit of AI is the ability to handle large amounts of data that can be quickly accessed as knowledge by the user. In other fields, there is an indication of opportunities for AI to leverage big data to obtain insights and develop strategies for managing specific diseases, including opioid use disorders.^10^ Another example of recognition and interpretation was offered by Liu et al, in which AI and orthopedic surgeons correctly identified a similar number of tibial plateau fractures (accuracy 0.91 vs 0.92).^11^ These use cases could improve efficiency and accuracy in diagnosis and treatment, ultimately leading to better patient outcomes. Other real‐world implications of AI in the field of otolaryngology include its use to augment histopathological diagnostics in head and neck neoplasms through the use of AI image analysis tools,^12^, ^13^ prediction of sensorineural hearing loss outcomes,^14^ improve treatment of obstructive sleep apnea,^15^ and many more. In these instances, machine learning was integrated into a commercially available hearing aid, which “learned” the individual's preferred gains from their volume control adjustments and gradually changed the default settings at power‐up. These adjusted and preferred settings by the user were at significantly different power levels than their prescribed target.^14^ As noted by Brennan et al, AI and machine learning have been used for OSA treatment by predicting treatment outcomes of different treatment options, evaluating treatments as they are administered, and personalizing treatments to the individual patient with improving understanding of underlying mechanisms of OSA.^15^ Recent studies have also evaluated the use of AI in otolaryngology education, such as the use of LLMs to answer patient questions.^16^

Additionally, AI can provide personalized learning experiences tailored to individual student needs and abilities.^17^ This may help improve student engagement and knowledge retention, leading to more effective learning, although it will take more research to determine whether, and to what degree, this is true.^18^

The rapid improvement in GPT4's performance can be attributed to the fact that it is “trained” on 10 times the amount of data as ChatGPT 3.5 and that OpenAI's pre‐trains the transformer neural network (the training period on which GPT4 “learns” in an unsupervised process to predict the next word in a sequence given the previous words).^19^ It is multimodal and can accept and produce text and image inputs and outputs. This allows it to have better context comprehension and succeed in more complex tasks such as analyzing an image and contextualizing it with a question stem.^19^ As data processing and analysis capabilities rapidly advance, it is important that physicians are aware of these developments and leverage the capabilities of these powerful and readily accessible tools.

### Limitations

The limitations of this study, specifically, are the lack of visual identification, interpretation, and integration within the questions. 26.8% of the questions contained an image, figure, or chart that resulted in the exclusion of these questions. Naturally, the real WQE contains images, and many aspects of otolaryngologic care require interpretation and analysis of images, radiographs, and tactile feedback such as a physical examination. Additionally, images may have contained more questions that required a higher application of knowledge or more challenging questions for the LLM's that may have potentially biased the results. Although images are an important part of otolaryngology, ChatGPT 3.5's input is exclusively text. With ChatGPT4 being able to process images with the use of open‐source plug‐ins and recently obtaining image analysis capabilities, AI use in image analysis is rapidly improving, with future iterations potentially evaluating images at a high level. However, as a preliminary analysis, this study of text‐based questions alone was sufficient to demonstrate the capacity of these LLM's in this context, as well as their shortcomings, while highlighting the rapid advancement between ChatGPT 3.5 and GPT4. It is also important to note that the questions bank which served as the source of the database is not derived from an official otolaryngology board examination, but rather an amalgam of representative questions. General limitations that apply to any AI model include the data sets they are trained on, which may incur, perpetuate, or even amplify existing societal biases or inequalities, and they could contain inaccurate or outdated information. Finally, limitations specific to these LLM's are based on its training using broad nonspecific information, and that it excels in specific fields of summation, translation, and text generation. However, they may not understand context or nuance‐specific language in knowledge‐specific areas which could lead to inaccurate or misleading responses.

## Implications for Practice

Although ChatGPT 3.5 likely would not have passed the ABOto WQE, it provided insightful and well‐constructed explanations for the correct answers, and it achieved results consistent with the 8th percentile of otolaryngology residents. Meanwhile, ChatGPT4 demonstrated the rapid advancement of AI, boasting a much higher correct response rate on text‐based questions (86.0%) compared to its predecessor (45.2%). It also answered 64.7% of image‐based questions correctly, bringing the overall correct answer rate to 80.3%, still slightly above the average resident score of 76.38% on the total question set. Overall, these findings indicate the potential of AI to assist and enhance medical education and health care in the future, while also underscoring the rapid improvement of the capabilities of this technology. As advancements in AI technology continue, particularly in areas such as image‐based recognition, interpretation, and specific‐domain applications of knowledge, it may be that AI‐assisted medical education, diagnosis, and treatment planning become commonplace in the medical and surgical landscape.

## Author Contributions

**Evan A. Patel**, drafted, edited, and approved the final manuscript and take full responsibility for its content; **Lindsay Fleischer**, drafted, edited, and approved the final manuscript and take full responsibility for its content; **Peter Filip**, drafted, edited, and approved the final manuscript and take full responsibility for its content; **Michael Eggerstedt**, drafted, edited, and approved the final manuscript and take full responsibility for its content; **Michael Hutz**, drafted, edited, and approved the final manuscript and take full responsibility for its content; **Elias Michaelides**, drafted, edited, and approved the final manuscript and take full responsibility for its content; **Pete S. Batra**, drafted, edited, and approved the final manuscript and take full responsibility for its content; **Bobby A. Tajudeen**, drafted, edited, and approved the final manuscript and take full responsibility for its content.

## Disclosures

### Competing interests

None.

### Funding source

None.
